# Molecular changes during TGF *β*‐mediated lung fibroblast‐myofibroblast differentiation: implication for glucocorticoid resistance

**DOI:** 10.14814/phy2.13669

**Published:** 2018-04-13

**Authors:** Jean‐Didier Breton, Déborah Heydet, Lora M. Starrs, Tim Veldre, Reena Ghildyal

**Affiliations:** ^1^ Respiratory Virology Group, Centre for Research in Therapeutic Solutions Faculty of Science and Technology University of Canberra Canberra Australia; ^2^ ANU Medical School The Australian National University Canberra Australia; ^3^ Present address: The John Curtin School of Medical Research Australian National University Canberra Australia

**Keywords:** Airway remodeling, glucocorticoid resistance, myofibroblast, TGF*β*, transdifferentiation

## Abstract

Airway remodeling is an important process in response to repetitive inflammatory‐mediated airway wall injuries. This is characterized by profound changes and reorganizations at the cellular and molecular levels of the lung tissue. It is of particular importance to understand the mechanisms involved in airway remodeling, as this is strongly associated with severe asthma leading to devastating airway dysfunction. In this study, we have investigated the transforming growth factor‐*β* (TGF *β*, a proinflammatory mediator)‐activated fibroblast to myofibroblast transdifferentiation pathway, which plays a key role in asthma‐related airway remodeling. We show that TGF *β* induces fibroblast to myofibroblast transdifferentiation by the expression of *α* SMA, a specific myofibroblast marker. Furthermore, Smad2/Smad3 gene and protein expression patterns are different between fibroblasts and myofibroblasts. Such a change in expression patterns reveals an important role of these proteins in the cellular phenotype as well as their regulation by TGF *β* during cellular transdifferentiation. Interestingly, our data show a myofibroblastic TGF *β*‐mediated increase in glucocorticoid receptor (GR) expression and a preferential localization of GR in the nucleus, compared to in fibroblasts. Furthermore, the GR *β* (nonfunctional GR isoform) is increased relative to GR *α* (functional isoform) in myofibroblasts. These results are interesting as they support the idea of a GR *β*‐mediated glucocorticoid resistance observed in the severe asthmatic population. All together, we provide evidence that key players are involved in the TGF *β*‐mediated fibroblast to myofibroblast transdifferentiation pathway in a human lung fibroblast cell line. These players could be the targets of new treatments to limit airway remodeling and reverse glucocorticoid resistance in severe asthma.

## Introduction

Airway remodeling refers to the structural modifications of the normal architecture of the airway wall. Such tissue reorganization involves changes in the composition and the organization of its cellular and molecular constituents contributing to the thickening of airway walls (Kuwano et al. 1993; James 1997). Airway remodeling has been attributed to repetitive injury to the airway wall arising from cycles of inflammation such as during repair, and asthma (Ganesan and Sajjan 2013). One of the main structural changes observed during remodeling includes the transdifferentiation of fibroblasts into myofibroblasts. Since myofibroblasts are practically absent in normal airways, fibroblast to myofibroblast transdifferentiation is therefore one of the key events contributing to the chronic sequel of asthma (Hackett et al. 2009), ultimately leading to permanently impaired pulmonary function (Pascual and Peters 2005).

Transforming growth factor‐*β* (TGF*β*) exerts multiple essential biological functions. Importantly, it plays a major role in lung function and in the pathogenesis of pulmonary diseases. Dysregulations in TGF*β* cellular pathway have been implicated in numerous chronic lung conditions, including asthma. Indeed, its expression has been shown to be increased in chronic lung diseases (Vignola et al. 1997). Interestingly, studies suggest that TGF*β* expression is upregulated in asthmatic lungs (Redington et al. 1997; Vignola et al. 1997; Tillie‐Leblond et al. 1999; Chu et al. 2000). In the lung, TGF*β* is involved in airway remodeling (Desmouliere 1995; Doherty and Broide 2007). It has been shown to promote fibroblast to myofibroblast transdifferentiation and to trigger their proliferation (Michalik et al. 2009). Therefore, taken together, these studies suggest that TGF*β* is tightly associated with asthma‐related airway remodeling.

The major signaling pathway of TGF*β* is through its transmembrane serine/threonine kinase receptor (Nakao et al. 1997; Attisano and Wrana 2000; ten Dijke et al. 2000; Massague and Chen 2000; Massague and Wotton 2000; Shi and Massague 2003). Activated TGF*β* receptor stimulates the phosphorylation of cytoplasmic receptor‐regulated Smad proteins. The activated Smad2 and Smad3 proteins form complexes, which subsequently translocate and accumulate in the nucleus, where they regulate the transcription of target genes. Recent studies show that TGF*β*‐induced transdifferentiation of fibroblasts into myofibroblasts is characterized by the induction of alpha‐smooth muscle actin (*α*SMA) by the Smad complex (Desmouliere et al. 1993; Ronnov‐Jessen and Petersen 1993; Roy et al. 2001). Therefore, *α*SMA is commonly used to define the myofibroblastic phenotype, whereas vimentin, an intermediate cytoplasmic filament protein, is routinely used as nonspecific marker for both fibroblasts and myofibroblasts (McAnulty 2007).

Glucocorticoids are the most potent anti‐inflammatory drugs used in the treatment of inflammatory diseases such as asthma (Donohue and Ohar 2004; Raissy et al. 2013). Their cellular effects are mediated by the glucocorticoid receptor (GR). In the absence of ligand, GR is primarily cytoplasmic; while following ligand binding, it translocates to the nucleus, where it induces or represses the transcription of specific target genes (Galon et al. 2002; Lu et al. 2007; Ren et al. 2012). Glucocorticoids are the most effective anti‐inflammatory medication for the treatment of asthma. Unfortunately, asthma resistance to glucocorticoids has been observed and such glucocorticoid resistance is a major barrier to the treatment of severe asthma (Holgate and Polosa 2006; Jarjour et al. 2012).

Numerous studies have proposed a role for GR isoforms in steroid resistance (Hamid et al. 1999; Sousa et al. 2000). The two major GR isoforms, GR*α* and GR*β*, are generated through alternative splicing. While GR*α* mediates the actions of glucocorticoids leading to GR‐mediated cellular response, GR*β* does not bind glucocorticoid agonists, limiting glucocorticoids‐mediated target gene transcription regulation (Bamberger et al. 1995; Pujols et al. 2007). Therefore, when both GR splice variants are coexpressed, GR*β* acts as a dominant negative inhibitor and antagonizes the activity of GR*α* (Bamberger et al. 1995; Yudt et al. 2003). This ability of GR*β* to inhibit GR*α* activity suggests that increased levels of GR*β* could lead to glucocorticoid resistance (Sousa et al. 2000; Boardman et al. 2014).

Given the importance of fibroblast to myofibroblast transdifferentiation in airway diseases, we set out to investigate the TGF*β* molecular pathways in fibroblasts and myofibroblasts using a human lung fibroblast cell line (WI‐38). Here we use WI‐38 treated with TGF*β* to induce fibroblast to myofibroblast transdifferentiation or left untreated to demonstrate that TGF*β* signaling pathway is affected in myofibroblasts. TGF*β*‐treated myofibroblasts exhibit decreased Smad3 gene and protein expression. We also provide molecular evidence for a role of GR*β* in glucocorticoid resistance in asthma‐like cells.

## Materials and Methods

### Antibodies

The primary antibodies for the following proteins were used for western blot analysis and immunofluorescence (IF): *α*SMA (ab7817, Abcam), *α*/*β*‐Tubulin (2148, Cell Signaling Technology), GR (sc‐8992, Santa Cruz Biotechnology), Smad2/3 (sc‐133098, Santa Cruz Biotechnology), Vimentin (sc‐5565, Santa Cruz Biotechnology).

### Cell culture

Human embryonic lung fibroblasts, WI‐38 cell line, were obtained from the European Collection of Cell Cultures (ECACC, Ref No 90020107). Cells were grown in DMEM (Life Technologies, Carlsbad, CA) supplemented with 10% fetal bovine serum (FBS; Bovogen, Australia) and antibiotics (penicillin, streptomycin, neomycin; Life Technologies) at 37°C in a humidified atmosphere of 5% CO_2_.

### TGF*β*1 treatment

At day 0 cells were either seeded in 35 mm Petri dishes (for day 1 collection, D1) or maintained in 25 cm^2^ flasks for later collection (when myofibroblast transdifferentiation was obtained). Cells were incubated in DMEM with 10% FBS, antibiotics and 2–5 ng/mL TGF*β*1 (R&D systems, Minneapolis, MN). TGF*β*1 supplemented media was changed every second day until collection. Myofibroblast phenotype was confirmed by western blot and immunofluorescence for *α*SMA. Once transdifferentiation was achieved cells were collected for further analysis with respective untreated controls (see below for determination of fibroblast to myofibroblast transdifferentiation). Samples were labelled as follow D1 or D20 WI‐38 +TGF*β* (with) or ‐TGF*β* (without).

### Western blot analysis

Overnight confluent cultures of WI‐38 ± TGF*β*1 were collected for protein extraction at the specified time points. Whole‐cell lysates were collected in RIPA buffer (150 mmol/L NaCl, 1% Triton X‐100, 0.5% sodium deoxycholate, 0.1% SDS, 50 mmol/L Tris, with protease and phosphatase inhibitors). Then, protein extracts were heated at 90°C for 5 min in Laemmli buffer prior to SDS‐PAGE. Cell lysates were subjected to SDS‐polyacrylamide electrophoresis followed by transfer to nitrocellulose membranes in Tris‐glycine‐ethanol buffer (25 mmol/L Tris‐HCl, 192 mmol/L glycine, 20% ethanol). Blots were stained with Ponceau S (Sigma, St Louis, MO) to confirm proteins transfer and then blocked for 1 h in PBS Odyssey Blocking Buffer containing 0.1% Tween 20 or 2.5% skim milk in PBS‐T (10 mmol/L Na_2_HPO_4_, 1.7 mmol/L KH_2_PO_4_, 2.7 mmol/L KCl, 137 mmol/L NaCl, 0.1% Tween 20), prior to incubation with different primary antibodies diluted in PBS Odyssey Blocking Buffer containing 0.1% Tween 20 or 2.5% skim milk in PBS‐T overnight at 4°C. Primary antibodies were detected using LI‐COR IRDye Infrared Dye (1:15000) or horseradish peroxidase (HRP; 1:5000) secondary antibodies. Detection of HRP bound antibodies was performed with enhanced chemiluminescence (Perkin Elmer, Waltham, MA). Blots were visualized using the Odyssey Fc Infrared Imager (LI‐COR Biotechnology, NE) and quantified by densitometry using Image Studio software (LI‐COR, NE). Results are expressed as relative to corresponding *α*/*β*‐tubulin and normalized to D1 WI‐38 ‐TGF*β*, which constitutes our control group. Where required, blots were stripped (2% SDS, 62.5 mmol/L Tris‐HCl, 114.4 mmol/L *β*‐mercaptoethanol) at 50°C for 10 min, washed and reprobed.

### mRNA analysis by real‐time PCR

Total RNA was extracted from overnight confluent cultures of WI‐38 ± TGF*β*1 using TRI Reagent (Sigma), first‐strand cDNA synthesized from 1 *μ*g RNA (High‐Capacity cDNA Reverse Transcription Kit, Life Technologies) and cDNA for *α*SMA, GAPDH, GR, Smad2, Smad3 (see Table 1 for description of primer sequences) estimated by semiquantitative real‐time PCR using Bio‐Rad CFX96 (Bio‐Rad, CA). Data were analyzed using CFX Manager Software version 3.1 (Bio‐Rad), and is presented relative to GAPDH and normalized to D1 WI‐38 ‐TGF*β*.

**Table 1:** Primer sequences used for semiquantitative real‐time PCR analyses

Gene	Forward primer	Reverse primer
*α*SMA	TTATGTTTGAGACTTTCAATGTC	GTCCAGAGGCATAGAGAG
GAPDH	TGGTATGACAACGAATTTGG	TCTACATGGCAACTGTGAGG
GR	GATGTCATTATGGAGTCTTAACTT	TTGTGCTGTCTACCTTCC
Smad2	GCTTTACAGACCCATCAAAT	CCTCTTCCTATATGCCTTCT
Smad3	CTACCAGTTGACCCGAAT	CAGTCTGTCTCCTGTACTC

### Immunofluorescence (IF)

Overnight confluent cultures of WI‐38 ± TGF*β*1 were grown on glass coverslips. Cells were fixed in 4% formaldehyde for 10 min and blocked/permeabilized in 2% BSA containing 0.5% Triton X‐100 for 10 min. Cells were then incubated with primary antibodies overnight at 4°C and bound antibodies were detected by Alexa Fluor conjugated secondary antibodies (1:1000; Life Technologies). Coverslips were mounted on slides in ProLong Gold reagent with DAPI (Life Technologies). Samples were examined under a Nikon Ti Eclipse confocal laser‐scanning microscope (CLSM) with Nikon 60x/1.40 oil immersion lens (Plan Apo VC OFN25 DIC N2; optical section of 0.5 *μ*m) and the NIS Elements AR software (Nikon Corporation, Japan). ImageJ 1.48v shareware (courtesy of Wayne Rasband, NIH) was used to analyze the digital images to determine the relative intensity of fluorescence in the nucleus (Fn) and in the cytoplasm (Fc) in order to estimate the nucleus‐cytoplasm fluorescence ratio (Fn/c ratio) after the subtraction of background fluorescence. Fn and Fc intensities were measured from 40 cells in each experimental condition.

### Myofibroblast determination

It is well accepted that cell expression of *α*SMA is a specific marker of fibroblast to myofibroblast transdifferentiation, whereas vimentin is considered as a nonspecific fibroblast and myofibroblast marker (McAnulty 2007). Therefore, we used *α*SMA and vimentin antibodies to determine fibroblast to myofibroblast transdifferentiation by IF and western blot analysis. In this study, we defined fibroblast to myofibroblast transdifferentiation using the following criteria: (1) coexpression of *α*SMA and vimentin should be observed in cells using IF staining and (2) TGF*β*‐treated samples with positive *α*SMA IF staining should be associated with at least a fivefold increase in *α*SMA expression.

### Statistical analyses

Data are presented as mean ± SEM for all experiments. From these experiments, statistical analysis was performed setting the level of statistical significance at *P* < 0.05. Statistical analysis was performed using two‐way ANOVA followed by correction for multiple comparisons using Sidak's test in Prism 6 software (GraphPad software, CA). Data were grouped by treatment and time.

## Results

### Effect of TGF*β* treatment on *α*SMA and vimentin expression in human lung fibroblasts and myofibroblasts

**Figure 1:**
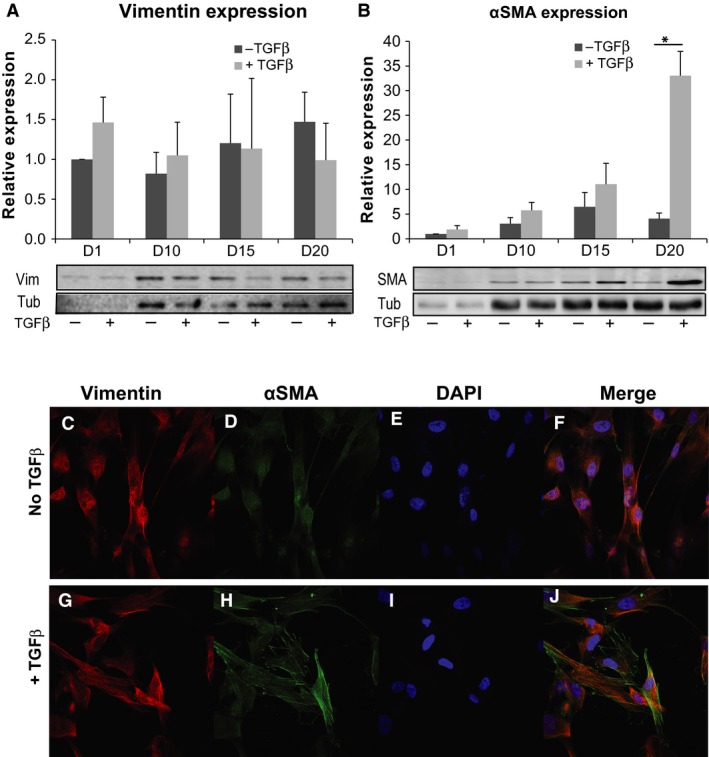
TGF *β* induces fibroblast to myofibroblast transdifferentiation. (A and B) Graphs show the relative protein expression for vimentin and *α* SMA in TGF *β*‐untreated and ‐treated cells at indicated timepoints. Representative western blots for vimentin and *α* SMA in the different experimental conditions are shown below the graphs; Tub = tubulin used as loading control. Note the significant increase in *α* SMA protein expression at D20 in B. (C–J) Immunofluorescence staining for vimentin, *α* SMA and DAPI in TGF *β*‐untreated (top) and ‐treated (bottom) cells. +TGF *β*: TGF *β*‐treated cells; ‐ TGF *β*: TGF *β*‐untreated cells; *P < 0.05; data are mean ± SEM from three independent experiments.

TGF*β* plays a key role in airway remodeling, partly due to its capacity to induce fibroblast to myofibroblast transdifferentiation. Therefore, we first examined that TGF*β* treatment could induce such a transdifferentiation in WI‐38 cell line. Vimentin protein expression was measured at all timepoints (D1, D10, D15, and D20) and no significant change in its expression was observed through the treatment period (Two‐way ANOVA: *P* = 0.85; *n* = 3 in each group; Fig. 1A). Upon TGF*β* stimulation, WI‐38 showed a significant change in *α*SMA protein expression compared to untreated cells (Two‐way ANOVA: *P* = 0.0006; *n* = 3 in each group; Fig. 1B). Furthermore, higher *α*SMA protein expression was observed at all timepoints with an 8.1‐fold increase measured at D20 (Sidak's test, *P* < 0.001; *n* = 3 in each group; Fig. 1B), which is higher than the fivefold threshold we set to determine cell transdifferentiation.

In parallel, indirect IF was performed, where monolayers of WI‐38 ± TGF*β* grown on coverslips were fixed at D20 and probed for vimentin (Fig. 1C and G) in combination with *α*SMA (Fig. 1D and H) and counterstained with DAPI (Fig. 1E and I). Consistent with the western blot analysis, indirect IF showed that WI‐38 cells treated with TGF*β* induced fibroblast to myofibroblast transdifferentiation, indicated by the presence of positive *α*SMA staining in the treated cells (Fig. 1H and J) and the presence of vimentin (Fig. 1G) similar to the untreated cells (Fig. 1C). Taken together, these data suggest that treatment of WI‐38 with TGF*β* induced fibroblast to myofibroblast transdifferentiation at D20. Thus, following experiments will describe specific differences observed between fibroblasts (D1 WI‐38 ± TGF*β*, D20 WI‐38 ‐TGF*β*) and transdifferentiated myofibroblasts (D20 WI‐38 + TGF*β*).

### Effects of TGF*β* treatment on Smad2/3 expression in human lung fibroblasts and transdifferentiated myofibroblasts

**Figure 2:**
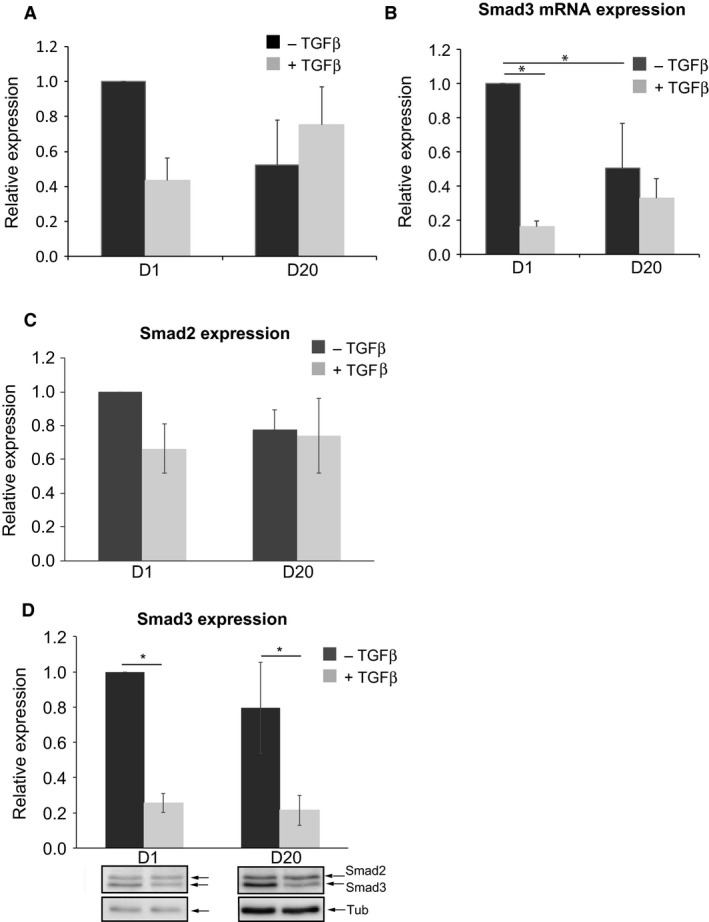
Characterization of Smad2 and Smad3 expression in fibroblast and myofibroblast during TGF *β* treatment. (A and B) Graphs show the relative expression of Smad2 and Smad3 mRNAs in fibroblast and myofibroblast in TGF *β*‐untreated and ‐treated cells. (C and D) Graphs show the relative expression of Smad2 and Smad3 proteins in fibroblast and myofibroblast in TGF*β*‐untreated and ‐treated cells. Representative western blot for Smad2/3 in the different experimental conditions is illustrated below (D) Tub = tubulin used as loading control**.** +TGF*β*: TGF*β*‐treated cells; ‐TGF*β*: TGF*β*‐untreated cells. Data shown are mean ± SEM from four independent experiments; *P < 0.05.

The mRNA levels of Smad2 and Smad3 were lower in TGF*β*‐treated fibroblast (D1 WI‐38 + TGF*β*) compared to untreated cells (D1 WI‐38 ‐ TGF*β*), with approximately two and sixfold decrease, respectively (Fig. 2A and B). Expression of Smad2 and Smad3 genes was lower in D20 WI‐38 ‐ TGF*β* compared to D1 WI‐38 ‐ TGF*β*, whereas it remained similar in D1 WI‐38 + TGF*β* compared to D20 WI‐38 + TGF*β*. Overall Smad2 mRNA levels were higher and Smad3 mRNA levels lower in myofibroblast (D20 WI‐38 + TGF*β*) compared to their respective control (D20 WI‐38 ‐ TGF*β*).

A similar pattern was observed in Smad2 and Smad3 protein expression with approximately 1.5 and a significant, fourfold decreased expression in TGF*β*‐treated fibroblast (D1 WI‐38 + TGF*β*) compared to untreated fibroblasts (D1 WI‐38 ‐ TGF*β*), respectively (Fig. 2C,D). Smad2 protein expression was unchanged in D20 WI‐38 ‐TGF*β* compared to D20 WI‐38 + TGF*β*, whereas Smad3 expression was significantly decreased 4.1‐fold in D20 WI‐38 + TGF*β* compared to D20 WI‐38 ‐ TGF*β* (Two‐way ANOVA, *P *= 0.009; Sidak's test, *P *= 0.036). These results indicate that TGF*β* treatment induces changes in Smad2 and Smad3 gene and protein expression patterns in both fibroblasts and myofibroblasts. These changes in protein and gene patterns might underlie a differential TGF*β*‐induced signaling pathway between fibroblast and myofibroblast.

### TGF*β*‐induced transdifferentiation upregulates GR*β* expression in human lung myofibroblasts

**Figure 3:**
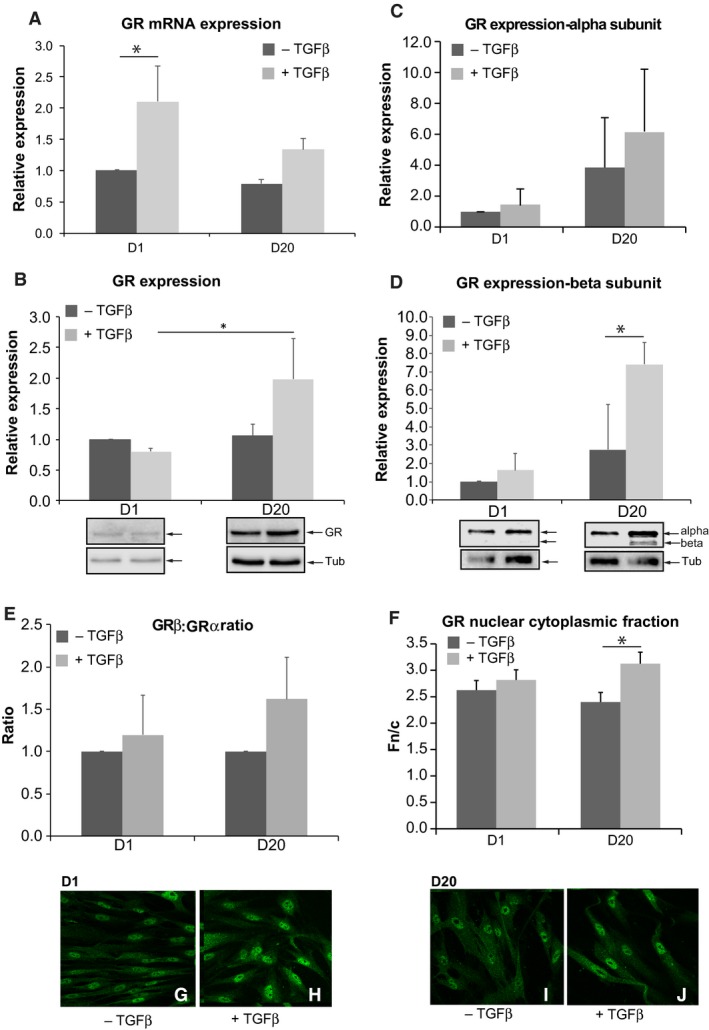
Glucocorticoid receptor expression in fibroblast and myofibroblast following TGF *β* treatment. (A) Graph shows the relative expression of glucocorticoid receptor (GR) mRNA in fibroblast and myofibroblast in TGF *β*‐untreated and ‐treated cells. +TGF *β*: TGF *β*‐treated cells; ‐TGF *β*: TGF *β*‐untreated cells. Data are mean ± SEM from three independent experiments; *P < 0.05. (B) Graph shows the relative expression of GR proteins in fibroblast and myofibroblast in TGF *β*‐untreated and ‐treated cells. Below the graph, representative western blots are illustrated for the different experimental conditions; Tub = tubulin used as loading control. +TGF *β*: TGF *β*‐treated cells; ‐ TGF *β*: TGF *β*‐untreated cells. Data are mean ± SEM from three independent experiments; *P < 0.05. (C and D) Graphs show GR alpha subunit (C) and beta subunit (D) in fibroblast and myofibroblast in TGF *β*‐untreated and ‐treated cells. The representative western blot for both isoforms is illustrated below (D). +TGF *β*: TGF *β*‐treated cells; ‐ TGF *β*: TGF *β*‐untreated cells. Data shown are mean ± SEM from three independent experiments; *P < 0.05. (E) Graph shows the ratio of GR *β*:GR *α* isoforms for each experimental condition. Note that TGF *β* increases GR *β* isoform. Data were normalized to D1 –TGF *β*. +TGF *β*: TGF *β*‐treated cells; ‐ TGF *β*: TGF *β*‐untreated cells. (F) Graph shows the relative fluorescence in the nucleus compared to that in the cytoplasm (Fn/c) of GR in both fibroblast and myofibroblast cells in different experimental conditions. Note the increased Fn/c at D20 in presence of TGF *β*. Each datapoint represents mean ± SEM of data from 40 cells; *P < 0.05. +TGF *β*: TGF *β*‐treated cells; ‐ TGF *β*: TGF *β*‐untreated cells. (G–J) Immunofluorescence images for GR expression in different experimental conditions: at D1–TGF *β* (G), at D1+TGF *β* (H), at D20–TGF *β* (I), at D20+TGF *β* (J).

There was a significant change in GR mRNA levels in TGF*β*‐treated fibroblasts (D1 WI‐38 + TGF*β*) compared to untreated fibroblasts but this was not reflected in the protein data (D1 WI‐38 ‐ TGF*β*; Fig. 3A and B). We observed a 2.0‐fold (nonsignificant) increase in GR mRNA and total protein expression in TGF*β*‐treated myofibroblasts (D20 WI‐38 + TGF*β*) compared to cells left untreated (D20 WI‐38 ‐ TGF*β*; Fig. 3A and B). Interestingly, a time‐dependent increase in GR protein levels was observed on TGF*β* treatment (D1 WI‐38 + TGF*β* compared to D20 WI‐38 + TGF*β*; Fig. 3B). GR is known to be expressed in two different isoforms (GR*α* and GR*β*) due to differential splicing. Thus, we then sought to establish which GR isoform was present in fibroblasts and myofibroblasts (±TGF*β*). We found that GR*α* was present in both fibroblasts and myofibroblasts, regardless of TGF*β* treatment (Fig. 3C). However, its expression was increased 1.3 and 1.5‐fold in TGF*β*‐treated cells (D1 WI‐38 + TGF*β* and D20 WI‐38 + TGF*β*, respectively) compared to their respective untreated controls (D1 WI‐38‐TGF*β* and D20 WI‐38 ‐ TGF*β*). Surprisingly, a 1.5‐fold increase in GR*β* expression was also observed in D1 WI‐38 + TGF*β* compared to the untreated group (D1 WI‐38 ‐ TGF*β*, Fig. 3D). Moreover, GR*β* was significantly expressed in D20 WI‐38 + TGF*β* compared to D20 WI‐38 ‐ TGF*β* (Fig. 3D), with a 3.0‐fold increase in the TGF*β*‐treated group compared to the untreated group at D20 (Two‐way ANOVA, *P *= 0.038, *n* = 3 in each treatment group; Sidak's test, *P *= 0.01). In summary, increased GR*α* and GR*β* protein levels were mainly observed in TGF*β*‐treated cells, regardless of the cell phenotype (Fig. 3C and D). GR*α* and GR*β* proteins are the active and the inactive isoforms of GR, respectively. Furthermore, their respective level in cells is thought to be involved in the glucocorticoids cell response. So, we then focused on the ratio of GR*α* and GR*β* isoforms under our experimental conditions. Interestingly, our results show that despite an increase in both GR*α* and GR*β* isoforms, GR*β* is the predominant isoform in TGF*β*‐treated cells at both D1 and D20 compared to their respective controls (D1: GR*β*:GR*α *= 1.2; D20: GR*β*:GR*α *= 1.6; Fig. 3E).

We then investigated if GR localization was altered in TGF*β*‐treated cells. GR activation occurs in the cytoplasm, whereas its cellular action occurs in the nucleus, via a translocation mechanism. As shown in Figure 3G–J, GR nuclear localization was increased in TGF*β*‐treated myofibroblast phenotype cells (D20 WI‐38 + TGF*β*) compared to untreated cells (D20 WI‐38 ‐ TGF*β*). Image analysis confirmed this observation, with a significant difference in the relative fluorescence in the nucleus compared to that in the cytoplasm (Fn/c) between treated and untreated cells at D20 (Two‐way ANOVA, *P* = 0.017, *n* = 40; Fig. 3F, I and J). No significant change was detected at D1 between TGF*β*‐treated and ‐untreated fibroblasts (Two‐way ANOVA: *P* = 0. 71; *n* = 40; Fig. 3F–H).

Taken together, these data show that TGF*β* treatment in WI‐38 cells increases both GR*α* and GR*β* expression irrespective of the time of treatment. Furthermore, we demonstrate that GR is predominantly localized in the nucleus of transdifferentiated myofibroblast upon TGF*β* treatment.

## Discussion

Fibroblast to myofibroblast transdifferentiation is one of the pivotal events contributing to chronic asthma sequels, which can result in severe impaired lung function. In this study, we focus our attention on the key molecular components involved in this transdifferentiation process. Our data describe for the first time that TGF*β* treatment of human lung fibroblast cell line (WI‐38) induces fibroblast to myofibroblast transdifferentiation. This mechanism is accompanied by an altered TGF*β* signaling pathway, involving at least Smad2/3. Importantly, we have also demonstrated that GR*β* expression is increased in myofibroblasts, which is potentially responsible for the glucocorticoid resistance observed in severe asthma.

### Airway remodeling in asthma

Airway remodeling is a key process leading to the progression of the symptoms associated with impaired pulmonary function in asthma (Lazaar and Panettieri 2003; Nihlberg et al. 2006; Yamauchi 2006). Airway remodeling is defined as a response of the airway wall to repetitive tissue injury leading to chronic inflammation and partial repair process (Nihlberg et al. 2006; Postma and Timens 2006; Yamauchi 2006). This remodeling is characterized by complex structural changes from cellular to molecular level, altering airway wall function and its constituents, among them: epithelium destruction, goblet cells hyperplasia, angiogenesis, and increase in basement membrane thickness due to extracellular matrix deposition ‐the so‐called subepithelial fibrosis (McDonald 2001; Postma and Timens 2006; Yamauchi 2006).

During asthma, inflammatory cells invade the airway wall and in combination with airway wall cells, they secrete inflammatory mediators like cytokines, including TGF*β*, which is described as playing an important role in regulating the airway remodeling process (Minshall et al. 1997; Panettieri 2003; Xu et al. 2003; Kay et al. 2004). Indeed, TGF*β* has numerous effects depending on cellular environment and cell condition (Makinde et al. 2007). Among the different responses to TGF*β* stimulation, the subepithelial layer fibrosis is one of the important processes occurring during airway remodeling. This fibrosis corresponds to the deposition of extracellular matrix (ECM) leading to the thickening of the basement membrane and the subepithelial layer.

### TGF*β* induces fibroblast to myofibroblast transdifferentiation

Myofibroblasts are almost absent in normal lung tissue. It has been shown that fibroblast to myofibroblast transdifferentiation is a critical event in the development of progressive lung fibrosis (Kuhn and McDonald 1991; Zhang et al. 1994; Phan 2002). Indeed, fibroblast to myofibroblast transdifferentiation is an important process leading to the subepithelial fibrosis (Makinde et al. 2007). TGF*β* is classically described as an important fibrogenic factor and it has been shown to trigger fibroblast to myofibroblast transdifferentiation in vitro as well as in vivo (Ronnov‐Jessen and Petersen 1993; Sime et al. 1997; Hashimoto et al. 2001). Furthermore, studies show that TGF*β* is involved in the pathogenesis of asthma. Indeed, TGF*β* upregulation in asthmatic airways triggers fibroblast to myofibroblast transdifferentiation (Redington et al. 1997; Vignola et al. 1997; Tillie‐Leblond et al. 1999; Chu et al. 2000).

Our data show that treatment with TGF*β* for 20 days induces the expression of *α*SMA in WI‐38 cell line (see Fig. 1). This intermediate filament protein is classically described as the principal feature in characterizing myofibroblastic phenotype (McAnulty 2007). Thus, we show that TGF*β* induces WI‐38 cell line transdifferentiation into myofibroblasts. Our data demonstrate that WI‐38 human lung fibroblast cell line can be used as a model of TGF*β*‐mediated myofibroblast transdifferentiation. This result is important as this model enables us to study molecular aspects of fibroblast to myofibroblast transdifferentiation‐related diseases in vitro, and more particularly TGF*β*‐associated lung conditions, like asthma.

### TGF*β*‐induced Smad3 downregulation in myofibroblast

The main intracellular signaling pathway of TGF*β* is well characterized. This pathway involves the activation of the transmembrane serine/threonine kinase TGF*β* receptor, leading to Smad2 and Smad3 phosphorylation (receptor‐regulated Smads or R‐Smads). They then form a heteromeric complex with Smad4 (common‐mediator Smad or Co‐Smad), which translocates to the nucleus where it regulates the transcription of specific target genes. This pathway is regulated by inhibitory Smads (including Smad7), which inhibit Smad2/3 phosphorylation during TGF*β* stimulation (Attisano and Wrana 2002; Shi and Massague 2003).

Our results demonstrate that TGF*β* differentially regulates Smad2 and Smad3 expression in WI‐38 cells. Indeed, Smad2 expression is not affected by TGF*β* at D20 (see Fig. 2A and C). Whereas, Smad3 gene and protein expressions are strongly downregulated following TGF*β* treatment at both D1 and D20 (see Fig. 2B and D).

Hu et al. (2003) have shown that TGF*β* treatment increases Smad3 expression in primary rat lung fibroblasts. Furthermore, Gu et al. (2007) found that TGF*β*‐induced *α*SMA gene upregulation is under the control of Smad3 in vitro. In our study, we show that *α*SMA gene expression is increased in TGF*β*‐treated cells (data not shown), similar to its protein expression (Fig. 1). This would suggest a role for Smad3 in fibroblast to myofibroblast transdifferentiation in WI‐38 cells. Interestingly, our data indicate a decrease in Smad3 expression following TGF*β* treatment (see Fig. 2D). This discrepancy with previous studies can be explained by the complex nature of the Smads signaling pathway. Indeed, *α*SMA gene promoter is also under the control of Smad2 in rodent vascular cells treated with TGF*β* (Corjay et al. 1989; Mack and Owens 1999). Our data do not indicate changes in Smad2 protein in TGF*β*‐transdifferentiated myofibroblast at D20. So, we can hypothesize that *α*SMA expression during TGF*β* treatment is mediated through Smad2. Furthermore, it is also possible that other TGF*β*‐activated signaling pathways may contribute to the transcriptional regulation of *α*SMA in myofibroblast. Indeed, it has been described that non‐Smad pathways, such as the phosphatidylinositol 3‐kinase (PI3K)/Akt pathway, participates in TGF*β*‐induced myofibroblast formation during epithelial to mesenchymal transition (Kim et al. 2006; Lamouille and Derynck 2007). Further experiments should be performed in order to clarify the cellular pathway(s) involved in the fibroblast to myofibroblast transdifferentiation and more precisely in the regulation of *α*SMA gene expression.

### Glucocorticoid receptor upregulation in TGF*β* induced myofibroblast

Glucocorticoids play an important role in many physiological functions essential for life, such as growth, inflammation, tissue repair, reproduction, metabolism, immune, cardiovascular, and nervous system functions (Kadmiel and Cidlowski 2013). In the cell, glucocorticoid responses are mediated by the activation and nuclear translocation of the cytoplasmic glucocorticoid receptor (GR). This activated receptor then regulates the transcription of target genes. In humans, alternate splicing of GR mRNA results in the synthesis of two functionally different isoforms. GR*α* is known to be the predominant isoform responsible for GR cellular response (Reichardt and Schutz 1998). While GR*β* is a dominant negative isoform of GR, whose presence induces inhibition of glucocorticoid activity (Bamberger et al. 1995). Thus, the cellular ratio of the two isoforms of GR could influence the cellular responses to glucocorticoids.

Studies show that GR is expressed in lung fibroblasts as well as myofibroblasts (Eickelberg et al. 1999; Baouz et al. 2005). Consistent with these studies, our data indicate that GR is expressed in WI‐38 cell line regardless of time and TGF*β* treatment. However, TGF*β* treatment induces a 2.0 and 1.5‐fold increase in GR mRNA and protein expression at D20, respectively (Fig. 3A and B). Our results demonstrate for the first time a differential expression of GR in WI‐38 fibroblast and myofibroblast. The expression of GR in cells, especially in fibroblasts and myofibroblasts, is important for cell homeostasis. Unfortunately, there is a lack of evidence correlating the observed increase in GR expression and fibroblast to myofibroblast transdifferentiation. Further investigation is required to elucidate the cellular mechanisms involved in GR regulation during TGF*β*‐induced fibroblast to myofibroblast transdifferentiation. Thus, our study leads to important questions on the role of GR and its regulation in myofibroblast‐related disease (such as asthma).

Our data show a significant increase in GR nuclear localization in TGF*β*‐transdifferentiated myofibroblast (D20, Fig. 3E–I). We also show that GR*β* isoform expression is strongly increased in transdifferentiated myofibroblast (D20, Fig. 3D). While we do not directly demonstrate the nature of the nuclear GR isoform, it is tempting to postulate that the observed increased GR in the nucleus is due to GR*β* isoform. Indeed, studies show that GR*β* isoform is constitutively localized in the nucleus of cells (Lewis‐Tuffin and Cidlowski 2006; Kino et al. 2009). In our study, the accumulation of GR*β* in the nucleus could be due to (1) a decrease in GR*β* degradation, (2) an increase in GR*β* expression and/or 3) an increase in nucleo‐cytoplasmic transport of this protein. Current research in the group is focused on assessing how TGF*β* regulates GR isoform expression and activity in the WI‐38 cell line.

All together, our data indicate that TGF*β*‐treated WI‐38 cell line is a relevant model to investigate the cellular and molecular mechanisms involved in fibroblast to myofibroblast transdifferentiation occurring in asthma.

Furthermore, our data indicate that GR*β* expression is predominant compared to GR*α* in TGF*β*‐transdifferentiated myofibroblast, as indicated by the increase in GR*β*:GR*α* ratio (D20, Fig. 3E). Interestingly, numerous studies demonstrate that increased GR*β* is associated with lung diseases, such as asthma (Hamid et al. 1999; Christodoulopoulos et al. 2000; Sousa et al. 2000; Pujols et al. 2004; Goleva et al. 2006). These studies also indicate that increased expression of the nonfunctional GR*β* isoform may account for glucocorticoid insensitivity in inflammatory diseases, among them asthma (Leung et al. 1997; Hamid et al. 1999; Sousa et al. 2000; Goleva et al. 2006). We can therefore hypothesize that TGF*β*‐transdifferentiated myofibroblasts from WI‐38 cell line present a resistance to glucocorticoid treatment. This cellular model represents a critical tool in the understanding of the underlying mechanisms of glucocorticoid insensitivity observed in steroid‐resistant severe asthma.

## Conclusion

In conclusion, the results of this study show that TGF*β* induces (1) fibroblast to myofibroblast transdifferentiation in a human lung fibroblast cell line, (2) decrease in Smad3 expression in myofibroblasts, 3) increase in GR*β* expression in myofibroblasts.

TGF*β* plays an important role in airway remodeling. This remodeling involves transdifferentiation of fibroblasts into myofibroblasts, a key event initiating tissue fibrosis observed in many lung diseases, such as asthma. This transdifferentiation mechanism implicates Smad3, a component of the TGF*β* signaling pathway. While Smad3 seems to be affected during WI‐38 myofibroblast transdifferentiation, the exact role of Smad3 in fibroblast to myofibroblast transdifferentiation is still not fully understood. Attention has been given to a potential role for GR to inhibit TGF*β* signaling pathway through a regulation of Smad3 (Song et al. 1999). So, further studies should focus on the interaction between TGF*β*, Smad3, GR and possible glucocorticoid insensitivity in WI‐38 human lung fibroblast cell line to provide greater insights in asthma‐related fibroblast to myofibroblast transdifferentiation and glucocorticoid resistance.

The human lung fibroblast WI‐38 cell line used in this study represents a promising model to study lung diseases and more particularly asthma and glucocorticoid‐resistant asthma in providing better understanding in the cellular and molecular aspects of such disease after myofibroblast transdifferentiation.

## Conflict of Interest

The authors declare no conflict of interest.
